# Attitudes toward a potential Alzheimer's Disease Vaccine in adults over the age of 50

**DOI:** 10.1002/alz70860_106360

**Published:** 2025-12-23

**Authors:** Craig T Curtis, Jenna Meador, Ruth Paiano, Kimberly Salazar, Cheryl Klenow, Kylie Webster, Ava C Curtis

**Affiliations:** ^1^ K2 Medical Research, The Villages, FL, USA

## Abstract

**Background:**

Vaccines directed against Alzheimer's Disease progression have entered clinical trials. Understanding attitudes and barriers to a potential Alzheimer's Disease vaccine are of vital importance as according to recent published literature, significant shifts in vaccine acceptance have occurred in older adults.

**Method:**

Brief survey administered to adults over age 50 (*n* = 168) exploring attitudes, acceptance or hesitancy of a potential Alzheimer's Disease vaccine

**Result:**

91% would accept an FDA approved vaccine against Alzheimer's Disease. Only 41% would accept an experimental vaccine in a clinical trial primarily due to concerns about unknown risks. Age was not a factor in acceptance across groups. Family history of or known acquaintance with Dementia increased overall acceptance of a vaccine against Alzheimer's Disease. The primary reason for lack of acceptance was lack of trust in the healthcare system, however, their trust increased if recommended by their primary healthcare provider.

**Conclusion:**

We have found that although these shifts have occurred towards non‐AD vaccines, adults in our survey were overwhelmingly accepting of a potential Alzheimer's Disease vaccine. However, the majority of adults would wait for FDA approval rather than accept an experimental vaccine in a clinical trial.

